# Circulating Cardiovascular Biomarkers 1–3 Years Following Hypertensive Pregnancy Disorders: Associations With Postpartum Cardiovascular Structure and Function

**DOI:** 10.1007/s43032-026-02171-y

**Published:** 2026-08-21

**Authors:** Kristina Klepp, Kjartan Moe, Daniel P. Jacobsen, Meryam Sugulle, Kristin Angel, Thomas G. von Lueder, Ralf Dechend, Anne Cathrine Staff

**Affiliations:** 1 Institute of Clinical Medicine, Faculty of Medicine, University of Oslo, Oslo, Norway; 2 Division of Gynaecology and Obstetrics, Ullevål, Oslo University Hospital, Oslo, Norway; 3 Norwegian Research Centre for Women’s Health, Oslo University Hospital, Oslo, Norway; 4 Department of Obstetrics and Gynaecology, Baerum Hospital, Vestre Viken Hospital Trust, Drammen, Norway; 5 Department of Cardiology, Oslo University Hospital, Oslo, Norway; 6 Oslo Cardiology, Carl Berner Clinic, Oslo, Norway; 7 Experimental and Clinical Research Center (Max-Delbrück Center for Molecular Medicine) and Medical Faculty of the Charité and Franz-Volhard Clinic, Helios-Klinikum, Helios Klinikum Berlin-Buch, Berlin, Germany

**Keywords:** Cardiovascular disease, Cardiovascular structure and function, Echocardiography, Hypertension, Hypertensive disorder of pregnancy, Postpartum, Preeclampsia

## Abstract

Hypertensive disorders of pregnancy (HDP) confer increased cardiovascular disease (CVD) risk postpartum. Screening methods targeting women with increased risk are lacking, limiting personalized follow-up. Circulating biomarkers may assist CVD risk stratification. We aimed to assess whether circulating biomarkers were associated with measures of cardiovascular structure and function following HDP and normotensive pregnancies (controls). We examined 275 women 1–3 years postpartum (176 following HDP and 99 parous controls). Circulating biomarkers (hs-CRP, non-HDL cholesterol, glucose, eGFR, cTnT, NT-proBNP, GDF-15, sHLA-G, SAA1) and cardiovascular function and structure measures were examined [pulse wave velocity (PWV), carotid intima-media thickness (CIMT) and echocardiography]. Group differences were tested by Mann-Whitney U test and chi-square. Associations were assessed by univariate regression analyses. A p-value < 0.05 was considered statistically significant. Women following HDP displayed significantly higher rates of hypertension (5% vs. 0%) and metabolic syndrome (7% vs. 1%) than controls. Circulating biomarker levels were similar between study groups. In univariate regression analyses for the prior HDP group, non-HDL cholesterol was significantly associated with PWV and CIMT. Additionally, glucose and hs-CRP were significantly associated with PWV. These associations were not reproduced for controls, except for non-HDL cholesterol which was significantly associated with CIMT. In conclusion, women following HDP and normotensive pregnancies had similar postpartum levels of circulating biomarkers, but circulating biomarkers were significantly associated with measures of arterial stiffness (PWV and CIMT) in women with prior HDP. Our findings support current guidelines recommending CVD follow-up after HDP. Non-HDL cholesterol, glucose and hs-CRP may possibly help refining personalized cardiovascular follow-up postpartum.

**Graphical Abstract:**

**Figure:**
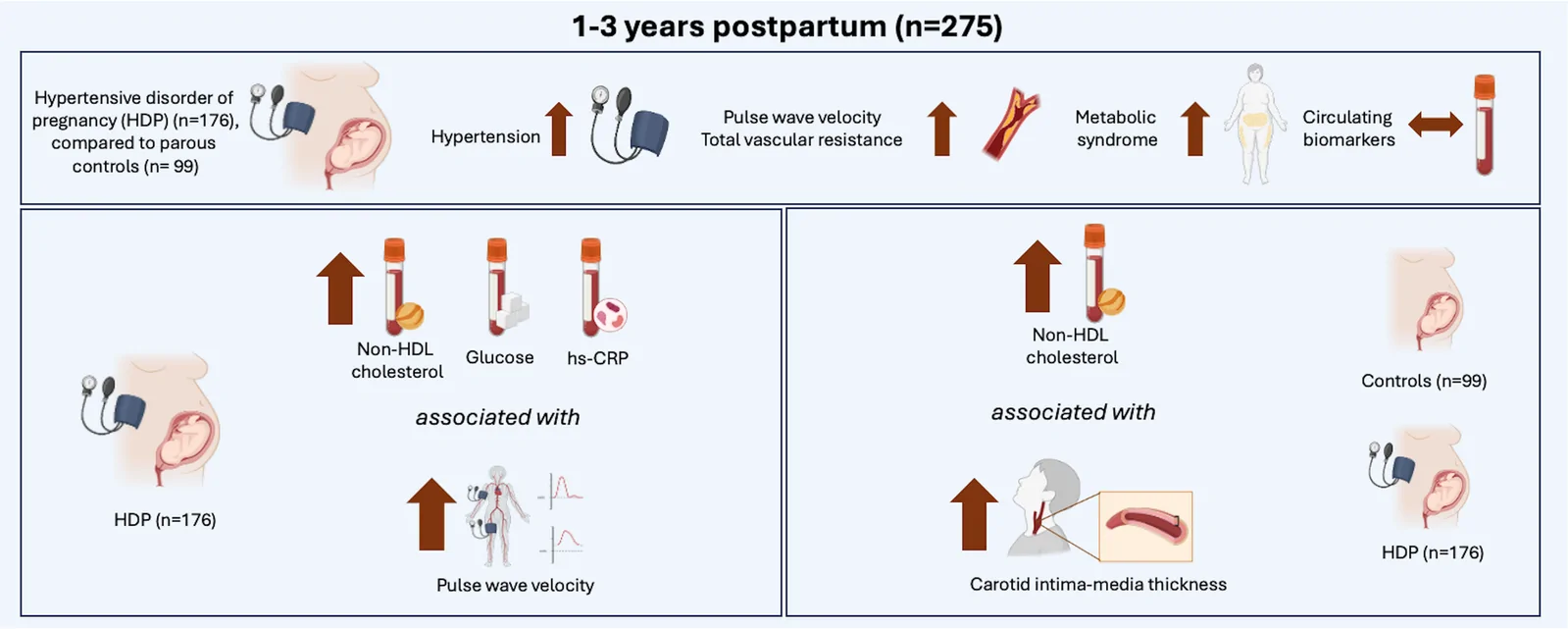

**Supplementary Information:**

The online version contains supplementary material available at https://doi.org/10.1007/s43032-026-02171-y.

## Introduction

Women with a history of hypertensive disorders of pregnancy (HDP) have a 2-8-fold higher risk of cardiovascular disease (CVD) later in life than women with prior normotensive pregnancies [1]. While global CVD mortality has decreased over the last 30 years, it remains a leading cause of mortality and disability adjusted life years (DALYs) worldwide [2]. HDP and CVD have several underlying risk factors in common, such as elevated body mass index (BMI), blood pressure, non-HDL cholesterol and the presence of diabetes [3, 4]. Hence, pregnancy can be seen as a physiological stress test for cardiovascular health later in life [5].

Within the first decade following HDP, women have an increased risk of developing hypertension [6]. Women with prior HDP also have an increased risk of persistent cardiovascular dysfunction postpartum, including more left ventricular remodeling and diastolic dysfunction [7–9], as well as increased pulse wave velocity (PWV) and carotid intima-media thickness (CIMT) [10]. Women with persistent hypertension after HDP are likely more prone to persistent abnormal cardiac remodeling postpartum [7, 11]. We have previously shown that adverse PWV, CIMT and cardiovascular phenotype evaluated by echocardiography can largely be explained by classical cardiovascular risk factors in clinically healthy women with a history of recent HDP [12, 13].

Circulating cardiovascular and inflammatory biomarkers are frequently used in clinical practice for risk stratification and diagnosing CVD. Circulating biomarkers associated with CVD include diabetes biomarkers (glucose) [3], estimated glomerular filtration rate (eGFR) [14], high-sensitive C-reactive protein (hs-CRP) [15], cardiac troponin T (cTnT) [16] and N-terminal pro-brain natriuretic peptide (NT-proBNP) [17]. Dyslipidemia is strongly associated with CVD, and non-HDL cholesterol (total cholesterol minus HDL cholesterol) is a marker associated with long-term CVD risk [3, 18]. Other circulating biomarkers have shown associations with CVD, but are less frequently used in clinical settings, including soluble human leukocyte antigen-G (sHLA-G) [19], serum amyloid A1 (SAA1) [20], and growth differentiation factor 15 (GDF-15) [21]. Women with prior HDP show dysregulated levels of several of these circulating biomarkers, including dyslipidemia [22], dysregulation of hs-CRP [23] and NT-proBNP [24] as well as increased risk of renal failure [25]. Our previously published studies suggest elevated levels of sHLA-G [26] and GDF-15 [27] after HDP compared to women with prior normotensive pregnancies, whereas reports of postpartum SAA1 are lacking.

The first decade following HDP is an important window of opportunity to identify women with increased CVD risk. However, no current risk calculator is suitable for cardiovascular risk evaluation in young women [28]. Hence, improved screening tools for CVD prediction are warranted to offer personalized intensified follow-up to women at highest risk. It is not established whether circulating biomarkers potentially can improve cardiovascular risk stratification postpartum. Cardiovascular assessments including PWV, CIMT and echocardiographic measures are surrogate markers for CVD. However, circulating biomarkers are more accessible and affordable for global populations than more extensive cardiovascular phenotyping. To investigate cardiovascular risk profiles following hypertensive disorders of pregnancies, we therefore aimed to investigate whether circulating cardiovascular and inflammatory biomarkers are associated with cardiovascular structural and functional markers one or three years postpartum.

## Methods

### Study Population

This cross-sectional study included 275 women from the HAPPY study [12, 28] at Oslo University Hospital, Ullevål, Norway. HAPPY included in total 451 postpartum women for a cardiovascular follow-up investigation one and/or three years after their index delivery at Oslo University Hospital (Supplementary Fig. 1). All women delivered a singleton baby and were ≥ 18 years at delivery. This substudy included women from the HAPPY cohort with an index pregnancy complicated by HDP and controls. Controls were women with normotensive and euglycemic index pregnancies, who delivered newborns without fetal growth restriction at term (≥ gestational week 37^0^), without HDP or premature birth in any prior pregnancy (Supplementary Fig. 1). Participants were further selected based on the availability of circulating biomarker and cardiovascular assessment data. Exclusion criteria included oocyte donation, diabetes, rheumatic, renal or malignant disease, and pregestational chronic cardiovascular disease. Women were also excluded if they were pregnant or breastfeeding at the time of follow-up, pregnant between index pregnancy and follow-up, or delivered an infant with overt chromosomal abnormalities.

In total, data from 275 women were analyzed. One-year follow-up data were analyzed from 210 participants, whereas three-year data were analyzed from the remaining 65 of the total cohort (Supplementary Figure S1 and Table 1). For study participants with both one- and three-year data available, follow-up data were analyzed from the time point with most relevant examinations available (e.g. PWV, CIMT and echocardiography). When similar amount of data was available from both follow-up times, the three-year data were analyzed. One woman participated in two subsequent pregnancies, in which only data from the first index pregnancy was analyzed.

**Table 1:** Clinical characteristics at 1-3-year follow-up for the study population (*n* = 275), by index pregnancy group. Data are presented as medians (interquartile range) for continuous variables. Categorical variables are presented as rates (n). Pregnancy complication groups (HDP, PE and GH) are compared to the control group. A p-value < 0.05 is marked with asterix (*)

Clinical data 1 or 3 years postpartum	Controls (*n* = 99)	HDP (*n* = 176)	*p*-value	PE (*n* = 132)	*p*-value	GH (*n* = 44)	*p*-value
Age (years)	35 (32–38)	35 (32–38)	0.48	35 (32–38)	0.58	35 (33–38)	0.47
Systolic BP (mmHg)	107 (101–111)	115 (107–123)	< 0.01*	114 (107–122)	< 0.01*	118 (110–126)	< 0.01*
Diastolic BP (mmHg)	63 (59–67)	71 (64–76)	< 0.01*	70 (64–75)	< 0.01*	73 (66–79)	< 0.01*
Mean arterial BP (mmHg)	77 (73–81)	86 (78–91)	< 0.01*	85 (78–90)	< 0.01*	89 (80–94)	< 0.01*
Normal BP (SBP < 120 and DBP < 80)	96.0% (95)	68.2% (120)	< 0.01*	72.7% (96)	< 0.01*	54.6% (24)	< 0.01*
Elevated BP (SBP 120–129 and DBP < 80)	2.0% (2)	13.6% (24)	< 0.01*	11.4% (15)	< 0.01*	20.5% (9)	< 0.01*
Stage 1 hypertension (SBP 130–139 or DBP 80–89)	2.0% (2)	12.5% (22)	< 0.01*	9.1% (12)	0.03*	22.7% (10)	< 0.01*
Stage 2 hypertension (SBP ≥ 140 and/or DBP ≥ 90)	0.0% (0)	5.1% (9)	0.03*	6.1% (8)	0.01*	2.3% (1)	0.31
Antihypertensive drug use	1.0% (1)	6.8% (12)	0.03*	9.1% (12)	< 0.01*	0.0% (0)	1.00
Recently diagnosed hypertension**	0.0% (0)	4.6% (8)	0.05	6.1% (8)	0.01*	0.0% (0)	-
Metabolic syndrome	1.0% (1)	7.4% (13)	0.02*	7.6% (10)	0.02*	6.8% (3)	0.09
Weight (kg)	64 (59–74)	67 (59–78)	0.25	66 (59–78)	0.37	69 (59–78)	0.24
Height (cm)	168 (164–172)	167 (163–171)	0.33	167 (163–171)	0.13	168 (165–173)	0.53
BMI (kg/m^2^)	22.9 (21.0–26.0)	24.1 (21.5–27.0)	0.07	24.3 (21.5–27.0)	0.07	24.0 (21.2–26.9)	0.33
Overweight (BMI ≥ 25 kg/m² and BMI < 30 kg/m²)	23.2% (23)	34.1% (60)	0.06	34.9% (46)	0.05	31.8% (14)	0.28
Obesity (BMI ≥ 30 kg/m²)	8.1% (8)	11.4% (20)	0.38	11.4% (15)	0.40	11.4% (5)	0.54
Premature CVD in 1st degree relative	15.2% (15)	31.3% (55)	< 0.01*	33.3% (44)	< 0.01*	25.0% (11)	0.16
Education > high school	77.8% (77)	76.1% (134)	0.18	73.5% (97)	0.22	84.1% (37)	0.34
White/not stated ethnicity	90.9% (90)	92.1% (162)	0.74	90.9% (120)	1.00	95.5% (42)	0.50
1 year visit data (of total 1 + 3-year visit data)	86.9% (86)	70.5% (124)	< 0.01*	67.4% (89)	< 0.01*	79.6% (35)	0.26
Time from pregnancy to follow-up (months)	13.9 (12.8–15.3)	14.3 (12.8–35.7)	0.11	14.4 (12.8–36.2)	0.0498*	14.0 (12.9–15.6)	0.92
Current smoking (yes)	11.1% (11)	6.8% (12)	0.22	6.1% (8)	0.17	9.1% (4)	1.00

The Mann-Whitney U test for continuous variables and Chi square or Fisher´s mid-p corrected test for categorical variables comparing pregnancy complication groups (HDP, PE and GH) to controls. Values are presented as medians (ranges) or rates (n). Blood pressures are categorized according to definitions from The American College of Cardiology/American Heart Association (ACC/AHA) Task Force on Clinical Practice Guidelines [31]. *BMI* body mass index, *BP* blood pressure, *CVD* cardiovascular disease, *GH* gestational hypertension, *HDP* hypertensive disorders of pregnancy (including both PE and GH patients), *PE* preeclampsia

**Recently diagnosed hypertension: includes women with new-onset hypertension after delivery (either diagnosed at this follow-up or started antihypertensive medical treatment after delivery)

Women with index pregnancies complicated by preeclampsia (PE), gestational hypertension (GH), or chronic hypertension with superimposed PE, were included in the HDP group. PE was defined as new-onset hypertension (systolic blood pressure (SBP) ≥ 140 mmHg and/or diastolic blood pressure (DBP) ≥ 90 mmHg) after gestational week 20 and at least one new-onset PE-associated feature after gestational week 20 (e.g., new-onset proteinuria, elevated transaminases, fetal growth restriction or stillbirth), according to the 2018 International Society for the Study of Hypertension in Pregnancy guidelines [29]. The PE group also encompassed women with eclampsia and/or HELLP (hemolysis, elevated liver enzymes and low platelets) in their index pregnancy. GH was defined as new-onset hypertension after gestational week 20, without any PE-associated feature [29]. Superimposed PE was defined as hypertension predating the pregnancy, or diagnosed before gestational week 20, and at least one new-onset PE associated feature after 20 weeks of pregnancy [29]. An offspring birthweight below the national 3rd percentile (in the absence of malformations or chromosomal abnormalities), adjusted for fetal sex and gestational age at delivery, was classified as fetal growth restriction (FGR) [30].

### Clinical Cardiovascular Assessments and Blood Sampling

In-patient hospital blood pressure (BP) was measured with a validated device (Dinamap Pro, 100VE, GE Medical Systems Information Technology, Inc. Milwaukee, WI, USA). One- and three-year follow-up BP assessments were conducted with an identical BP device as prior to delivery. According to clinical routine three consecutive measurements in the right upper arm, after 10 min rest in a supine position, with the head slightly elevated were conducted. Mean BP was calculated based on the last two measurements. We used blood pressure categories as defined by The American College of Cardiology/American Heart Association (ACC/AHA) Task Force on Clinical Practice Guidelines [31]. Normal BP was defined as SBP < 120 mmHg and DBP < 80 mmHg. Elevated BP was defined as SBP 120–129 mmHg and DBP < 80 mmHg. Stage 1 Hypertension was defined as SBP 130–139 mmHg and/or DBP 80–89 mmHg, and stage 2 Hypertension was defined as SBP ≥ 140 mmHg and/or DBP ≥ 90 mmHg.

At follow-up postpartum examination blood samples were collected. Sampling and analyses are previously described by us for similar cohorts [12, 26, 32, 33]. Measurements included circulating lipids, hs-CRP, cTnT, NT-proBNP, GDF-15, sHLA-G, SAA1, glucose and HbA1c. Non-HDL cholesterol was calculated by subtracting HDL cholesterol from total cholesterol. All women were fasting except three. For a randomly selected subgroup (*n* = 110), an oral glucose tolerance test (OGTT) (75 g glucose in 250–300 ml water) with a 2-hour measurement of serum glucose was conducted following a fasting serum glucose measurement.

Urine albumin/creatinine ratio was measured. Estimated glomerular filtration rate (eGFR) was calculated by the CKD-EPI formula [34]. Metabolic syndrome was defined according to the National Cholesterol Education Program (NCEP) Adult Treatment Panel III (ATP III) definition [35]. At follow-up, participants also completed questionnaires regarding general health, current smoking, socioeconomic factors, prior pregnancies (before the index pregnancy) and family history of CVD. A family history of CVD was registered if CVD or cardiovascular death occurred before 55 years of age for the patients´ father or before 65 years of age for the patient´s mother [36].

### Cardiovascular Assessment

One or more cardiovascular assessments were conducted for all included participants at one- or three-year follow-up. This encompassed PWV, CIMT and/or echocardiographic examinations. PWV was measured in 244 participants (89% of total), CIMT was measured in 258 participants (94% of total) and echocardiographic examinations were available for 95 participants (35% of total). In the total cohort, 81 participants (29%) underwent all three cardiovascular assessment examinations.

PWV and CIMT examinations were conducted by one examiner (KM), as previously described for a similar cohort at one year postpartum [12]. Two experienced cardiologists (KA and TGvL) conducted and interpreted all echocardiographic examinations at follow-up, as previously described by us [13]. Due to limited availability of trained examinators and labs, all cardiovascular assessments were performed on randomly selected subsets of the study participants. Echocardiography results have been published previously [13]. The echocardiographic measurements that were selected for the current regression analyses were total vascular resistance (TVR), relative wall thickness (RWT) and cardiac diastolic function (represented by the E/e´ ratio). Computed echocardiographic measurements are described in Supplementary Table S1, and age- and sex-specific reference ranges are presented in Supplementary Table S2.

### Statistical Analyses

Statistical analyses were conducted using the Statistical Package for the Social Sciences (PASW Statistics 30) and statistical software for data science (Stata Statistical Software: Release 18. College Station, TX, STATACorp LLC). Most continuous variables were not normally distributed. Results are therefore presented as medians and interquartile ranges, and group differences were tested by the Mann-Whitney *U* test. Categorical variables are presented as rates (%), and chi-square test and Fisher´s mid-*p* corrected test were used to test group differences. Univariate regression analyses were conducted to evaluate associations between circulating cardiovascular and inflammatory biomarkers and cardiovascular structure and function. Logarithmic transformations were conducted for all biomarkers, except for non-HDL cholesterol and eGFR, prior to regression analyses. To accommodate hs-CRP values of 0–1, hs-CRP was transformed as ln(CRP + 1). Sub-analyses for HDP (and HDP-subgroups; PE and GH) and controls were conducted to compare associations between study groups. Regression analyses for echocardiographic variables were not performed for the prior GH group, due to limited sample size (*n* = 6). A *p*-value < 0.05 was considered statistically significant.

## Results

In our final study cohort of 275 postpartum women (Supplemental Figure S1), 176 women were diagnosed with HDP during their index pregnancy (PE: *n* = 132; GH: *n* = 44), while the remaining 99 women had normotensive index pregnancies and served as controls. Postpartum clinical characteristics are summarized in Table 1, while postpartum circulating biomarker and cardiovascular assessment data are presented in Tables 2 and 3. Most study participants had higher education levels (> high school) and were of white ethnicity (77% and 91%, respectively), with no differences between study groups (Table 1).

**Table 2:** Circulating biomarkers at 1-3-year follow-up (*n* = 275), by index pregnancy group. Data are presented as medians (interquartile range) for continuous variables. A p-value < 0.05 is marked with asterix (*)

Circulating biomarkers 1 or 3 years postpartum	Controls (*n* = 99)	HDP (*n* = 176)	*p*-value	PE (*n* = 132)	*p*-value	GH (*n* = 44)	*p*-value
CIRCULATING LIPIDS							
LDL cholesterol (mmol/L)	2.73 (2.21–3.09)	2.74 (2.25–3.33)	0.47	2.77 (2.32–3.34)	0.31	2.61 (2.20–3.23)	0.82
HDL cholesterol (mmol/L)	1.53 (1.35–1.73)	1.51 (1.29–1.79)	0.64	1.50 (1.28–1.78)	0.55	1.55 (1.35–1.81)	0.996
Total cholesterol (mmol/L)	4.40 (3.80–4.90)	4.50 (4.00–5.00)	0.52	4.50 (4.10–5.00)	0.39	4.50 (3.90–4.90)	0.92
Non-HDL cholesterol (mmol/L)	2.89 (2.30–3.38)	2.86 (2.41–3.50)	0.58	2.89 (2.46–3.53)	0.38	2.71 (2.25–3.31)	0.69
Triglycerides (mmol/L)	0.68 (0.53–0.89)	0.70 (0.53–0.925)	0.72	0.71 (0.52–0.96)	0.78	0.70 (0.57–0.83)	0.71
KIDNEY FUNCTION							
Creatinine (µmol/L)	63.0 (58.0–68.0)	62.0 (56.0–68.0)	0.30	63.0 (57.0–68.0)	0.88	58.5 (55.0–64.5)	0.01*
eGFR (ml/min/1.73 m²)	112.67 (103.22–118.19)	113.86 (104.52–118.64)	0.43	113.13 (103.78–118.45)	0.91	115.12 (110.67–119.56)	0.06
Albumin/creatinine ratio (urine; mg/mmol)	0.3 (0.2–0.5)	0.5 (0.3–1.1)	< 0.01*	0.5 (0.3–1.0)	< 0.01*	0.5 (0.3–1.4)	0.01*
METABOLIC CONTROL							
Glucose (mmol/L)	4.7 (4.5–4.9)	4.7 (4.4–4.9)	0.61	4.7 (4.4–4.9)	0.42	4.7 (4.5–4.9)	0.72
Glucose 2 h after OGTT (mmol/L)	5.3 (4.6–5.9)	5.2 (4.5–6.0)	0.74	5.2 (4.5–6.0)	0.73	5.3 (5.0–5.6)	0.89
HbA1c (%)	5.0 (4.8–5.1)	5.0 (4.8–5.1)	0.59	5.0 (4.8–5.2)	0.74	4.9 (4.7–5.1)	0.09
INFLAMMATORY AND CARDIOVASCULAR BIOMARKERS							
hs-CRP (mg/L)	0.7 (0.0–1.2)	0.7 (0.0–1.7)	0.53	0.7 (0.0–1.7)	0.50	0.7 (0.0–1.7)	0.79
sHLA-G (µmol/L)	396.91 (250.28–577.81)	472.69 (278.59–596.50)	0.18	472.69 (253.10–598.22)	0.34	466.60 (343.41–591.34)	0.11
SAA1 (ng/ml)	2230.56 (1338.14–3326.28)	2194.02 (1295.00–3867.31)	0.93	2274.01 (1226.64–4281.85)	0.69	19999.89 (1434.25–3040.71)	0.52
GDF-15 (ng/L)	425.0 (400.0–509.0)	434.0 (400.0–534.5)	0.32	428.0 (400.0–535.0)	0.39	445.0 (400.0–517.0)	0.39
Cardiac troponin T (ng/L)	3 (3–3)	3 (3–3)	0.40	3 (3–3)	0.53	3 (3–3)	0.32
NT-proBNP (ng/L)	44 (28–64)	49 (31–72)	0.30	49 (32–74)	0.21	46 (29–71)	0.90

The Mann-Whitney U test for continuous variables comparing pregnancy complication groups (e.g., HDP) to controls. Values are presented as medians (interquartile ranges)

*eGFR* estimated glomerular filtration rate, *GDF-15* growth differentiation factor 15, *GH* gestational hypertension, *HDL, *high-density lipoprotein, *HDP* hypertensive disorders of pregnancy (including both PE and GH patients), *hs-CRP *high sensitivity C-reactive protein, *LDL *low-density lipoprotein, *NT-proBNP* N-terminal pro-brain natriuretic peptide, *OGTT* oral glucose tolerance test, *PE* preeclampsia, *SAA1 *serum amyloid A1, *sHLA-G* soluble human leukocyte antigen-G

**Table 3:** Measures of cardiovascular structure and function of the study population 1 or 3 years postpartum (*n* = 275), by index pregnancy group. P-values < 0.05 are marked with an asterix (*). n denotes number of women included in each investigation, by study group

Cardiovascular structure and function 1 or 3 years postpartum	Controls (*n* = 99)	HDP (*n* = 176)	*p*-value	PE (*n* = 132)	*p*-value	GH (*n* = 44)	*p*-value
ARTERIAL STRUCTURE AND FUNCTION							
PWV (m/s)	5.9 (5.5–6.4)	6.2 (5.7–6.8)	< 0.01*	6.2 (5.7–6.9)	< 0.01*	6.1 (5.8–6.8)	< 0.01*
n	89	154		116		38	
CIMT (mm)	0.45 (0.43–0.50)	0.47 (0.42–0.52)	0.10	0.46 (0.42–0.52)	0.39	0.49 (0.45–0.53)	< 0.01*
n	94	162		122		40	
**ECHOCARDIOGRAPHY VARIABLES**							
CARDIAC MORPHOLOGY/STRUCTURE							
SWd (cm)	0.7 (0.6–0.7)	0.7 (0.6–0.7)	0.20	0.7 (0.6–0.7)	0.16	0.7 (0.5–0.7)	0.97
n	34	60		54		6	
LVM (g)	100.2 (88.5–110.8)	103.1 (91.6–115.9)	0.46	104.9 (91.6–115.9)	0.38	98.3 (82.4–103.1)	0.75
n	34	60		54		6	
RWT	0.27 (0.24–0.30)	0.28 (0.25–0.32)	0.08	0.28 (0.26–0.33)	0.05	0.26 (0.23–0.30)	0.76
n	34	60		54		6	
FUNCTION							
TVR (dyne/s/cm^− 5^)	1365.8 (1239.6–1572.7)	1513.4 (1358.4–1726.4)	< 0.01*	1511.7 (1348.7–1696.7)	0.01*	1717.9 (1507.2–1759.2)	0.10
n	31	56		51		5	
SV (mL) (Doppler)	69 (63–76)	65 (60–71)	0.12	65 (60–71)	0.09	69 (62–74)	0.96
n	31	57		52		5	
CO (L/minute)	4.6 (4.2–5.0)	4.5 (4.0–5.2)	0.55	4.5 (4.0–5.2)	0.51	4.6 (4.5–4.8)	0.91
n	31	56		51		5	
LVEF (%) (biplane)	63 (62–66)	63 (61–65)	0.41	63 (61–65)	0.39	63 (62–67)	0.91
n	34	60		54		6	
DIASTOLIC FUNCTION							
E (cm/s)	76 (65–86)	77 (67–89)	0.48	78 (66–89)	0.47	77 (69–78)	0.85
n	33	59		54		5	
A (cm/s)	43 (38–50)	43 (39–53)	0.50	44 (39–53)	0.46	42 (40–47)	0.93
n	33	59		54		5	
E/A ratio	1.73 (1.50–2.09)	1.68 (1.47–2.05)	0.81	1.68 (1.45–2.05)	0.78	1.73 (1.62–1.77)	0.91
n	33	59		54		5	
E/e´ ratio	6.29 (5.62–6.82)	6.21 (5.78–7.32)	0.41	6.19 (5.62–7.32)	0.52	7.14 (6.21–7.15)	0.23
n	33	59		54		5	

The Mann-Whitney U test comparing pregnancy complication groups (HDP, PE and GH) to controls. Values are presented as medians (interquartile range). *CIMT* carotid intima-media thickness, *CO,* cardiac output, *LVEF* left ventricular ejection fraction, *LVM* left ventricular mass, *PWV* pulse wave velocity, *RWT* relative wall thickness, *SV* stroke volume, *SWd* septal wall diameter in diastole, *TVR* total vascular resistance

At the one- or three-year follow-up examination, the median age of the total cohort was 35 years, with no significant difference between study groups (Table 1). The group with a history of recent HDP displayed significantly higher blood pressure, compared to controls. This was also the case for the HDP subgroups (PE and GH), when compared separately to controls. All pregnancy complication groups also demonstrated significantly lower rates of normal blood pressure and higher rates of elevated blood pressure and stage 1 hypertension, compared to controls. Furthermore, women with prior HDP (as well as the PE subgroup) had significantly higher rates of stage 2 hypertension, compared to controls (Table 1). Women with prior HDP (as well as the PE subgroup) had significantly higher rates of metabolic syndrome, compared to controls. Additionally, median BMI was higher, and overweight and obesity were more frequently observed in the prior HDP group (as well as the PE subgroup) than in the control group, although the differences were not statistically significant (Table 1). A family history of CVD was significantly more prevalent in women with prior HDP and PE, compared to controls. The smoking rates were low overall but were non-significantly lower in the prior pregnancy complication groups, compared to controls (Table 1).

At follow-up, all women receiving current antihypertensive treatment were in the prior HDP group, except for one control participant whose medication was prescribed for migraine (Table 1). Furthermore, one participant (in the HDP group) used lipid-lowering medication at follow-up, and excluding this participant did not change the results. In the prior PE group, four study participants had chronic hypertension with superimposed PE. Despite the additional CVD burden, compared to PE alone, the main results remained consistent following sensitivity analyses without these women.

Index pregnancy characteristics (1–3 years prior to follow-up) are shown in Supplementary Table S3. Women with HDP in their index pregnancy had significantly higher prepregnancy BMI, delivered at significantly lower gestational age, delivered newborns with significantly lower birthweights and were significantly more often primiparous, compared to controls.

### Postpartum Circulating Biomarker Results

At follow-up, we observed no statistically significant differences in the median levels of circulating biomarkers between the prior HDP and HDP subgroups (PE and GH), and controls (Table 2). However, women with prior GH exhibited significantly lower median creatinine levels, compared to controls. Although not statistically significant, women with prior HDP (as well as the PE subgroup) exhibited higher median levels of sHLA-G, GDF-15 and NT-proBNP, compared to controls (Table 2). In our total cohort, 3 women (1 control and 2 HDP) did not meet fasting criteria. Excluding them from the lipid and glucose analyses did not alter the conclusions (data not shown).

The urine albumin/creatinine ratio at the one- or three-year postpartum control showed significantly higher levels in all prior pregnancy complication groups (HDP, PE and GH), compared to controls (Table 2). All groups with index pregnancy hypertension (HDP, PE and GH) also displayed significantly higher rates of urine albumin/creatinine ratio above reference range (normal; <3 mg/mmol), compared to controls. Two women with prior PE had an abnormal eGFR (< 90 ml/min/1.73 m²) and an elevated albumin/creatinine ratio (≥ 3 mg/mmol).

### Postpartum Cardiovascular Assessment Results

Assessments of vascular structure and function conducted 1–3 years postpartum revealed higher median pulse wave velocity (PWV) in the prior HDP, PE and GH groups, compared to controls (Table 3). Women with a history of recent GH also displayed significantly higher median levels of carotid intima-media thickness (CIMT), compared to controls (Table 3).

Echocardiographic assessments of cardiovascular structure and function revealed no significant differences between study groups, except for significantly higher total vascular resistance (TVR) in the prior HDP and PE groups, compared to controls (Table 3).

**Fig. 1:**
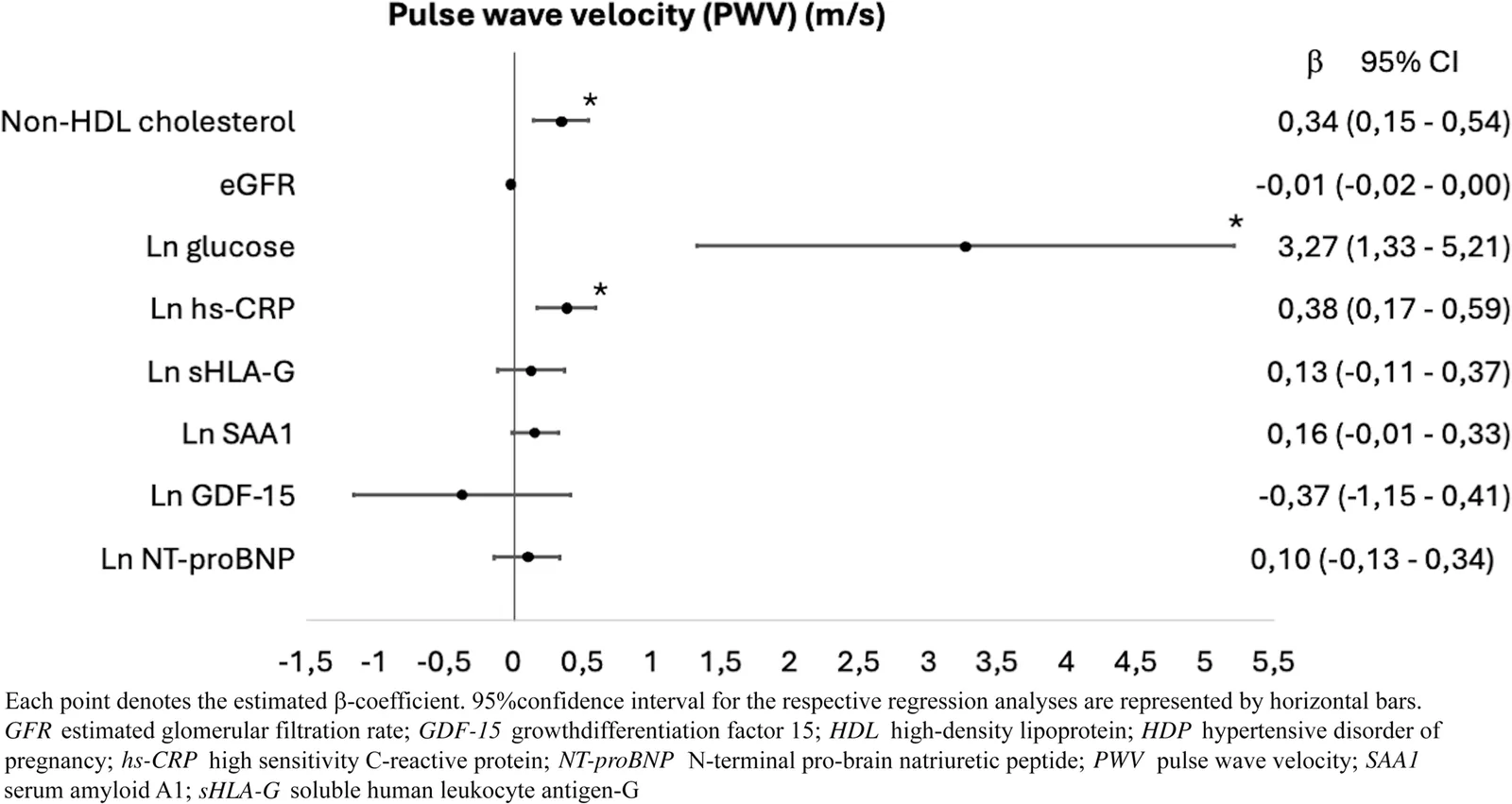
Regression coefficient plot for unadjusted regression analyses for the prior HDP group (*n*=176). Associations between circulating biomarkers and pulse wave velocity (PWV) at postpartum follow-up. Significant associations (*p*<0.05) are marked with asterix (*)

**Fig. 2:**
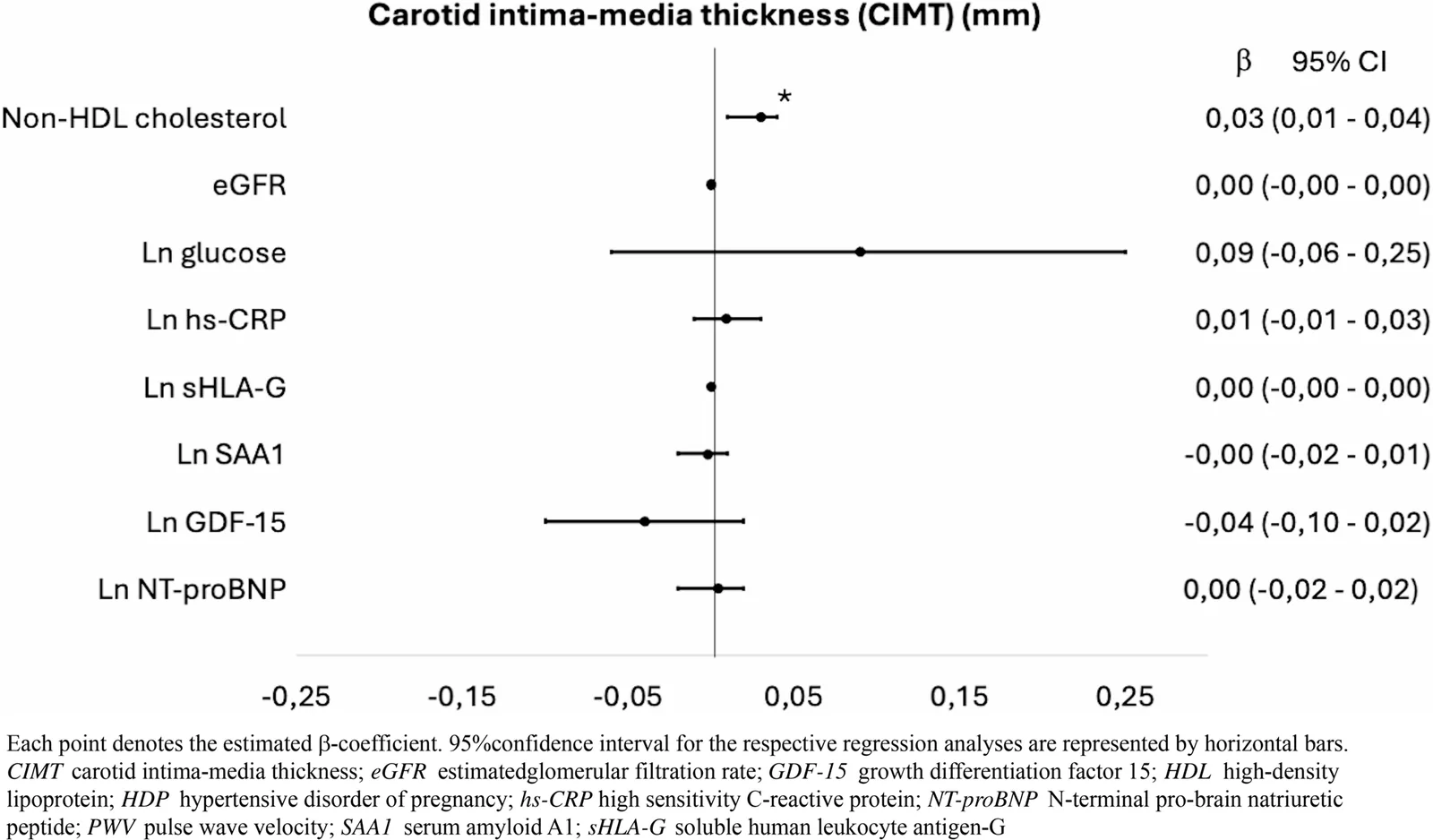
Regression coefficient plot for unadjusted regression analyses for the prior HDP group (*n*=176). Associations between circulating biomarkers and carotid intima-media thickness (CIMT) at follow-up. Significant associations (*p*<0.05) are marked with asterix (*)

In univariate regression analyses, non-HDL cholesterol was significantly associated with both PWV and CIMT for the prior HDP and PE groups (Tables 4 and 5; Figs. 1 and 2). Additionally, glucose and hs-CRP were significantly associated with PWV. Other circulating biomarkers did not show significant associations with measures of cardiovascular structure and function (Tables 4 and 5). For the prior GH group, glucose was significantly associated with PWV (Supplementary Table S5). Regression analyses for echocardiographic variables were not conducted due to the limited sample size (*n* = 6).

**Table 4:** Unadjusted regression analyses for the prior HDP group (*n* = 176). Associations between circulating biomarkers and cardiovascular structure and function at follow-up. Significant associations (*p* < 0.05) are marked with asterix (*). n denotes number of women included in each investigation, by study group

Circulating biomarkers	PWV (m/s)		CIMT (mm)		RWT		TVR (dyne/s/cm^− 5^)		E/e´ratio	
	b (95% CI)	*p*	b (95% CI)	*p*	b (95% CI)	*p*	b (95% CI)	*p*	b (95% CI)	*p*
Non-HDL cholesterol (mmol/L)	0.34 (0.15–0.54)	< 0.01*	0.03 (0.01–0.04)	< 0.01*	-0.01 (-0.03–0.00)	0.10	59.97 (-28.30–148.23)	0.18	-0.02 (-0.47–0.43)	0.93
n	154		162		60		56		59	
eGFR (ml/min/1.73 m²)	-0.01 (-0.02–0.00)	0.08	0.00 (-0.00–0.00)	0.43	0.00 (-0.00–0.00)	0.41	0.88 (-5.18–6.94)	0.77	-0.01 (-0.04–0.01)	0.35
n	153		161		60		56		59	
Ln glucose (mmol/L)	3.27 (1.33–5.21)	< 0.01*	0.09 (-0.06–0.25)	0.23	-0.01 (-0.13–0.12)	0.92	384.61 (-306.45–1075.67)	0.27	1.21 (-2.26–4.68)	0.49
n	150		158		60		56		59	
Ln hs-CRP (mg/L)	0.38 (0.17–0.59)	< 0.01*	0.01 (-0.01–0.03)	0.326	0.01 (-0.01–0.03)	0.201	-35.87 (-139.97–68.23)	0.49	-0.01 (-0.11–0.09)	0.810
n	154		161		59		55		58	
Ln sHLA-G (µmol/L)	0.13 (-0.11–0.37)	0.303	0.00 (-0.00–0.00)	0.530	-0.01 (-0.03–0.01)	0.255	19.69 (-93.91–133.30)	0.73	0.09 (-0.45–0.63)	0.73
n	144		161		59		55		58	
Ln SAA1 (ng/ml)	0.16 (-0.01–0.33)	0.06	0.00 ‡ (-0.02–0.01)	0.83	0.00 ‡ (-0.02–0.01)	0.70	20.39 (-63.16–103.94)	0.63	-0.06 (-0.46–0.35)	0.79
n	153		161		59		55		58	
Ln GDF-15 (ng/L)	-0.37 (-1.15–0.41)	0.35	-0.04 (-0.10–0.02)	0.17	-0.03 (-0.09–0.03)	0.29	-285.89 (-574.25–2.47)	0.05	-0.27 (-1.87–1.33)	0.74
n	146		161		59		55		58	
Ln NT-proBNP (ng/L)	0.10 (-0.13–0.34)	0.39	0.00 (-0.02–0.02)	0.74	0.00 (-0.02–0.02)	0.97	-32.91 (-141.74–75.93)	0.55	0.12 (-0.42–0.66)	0.66
n	147		162		60		56		59	

b: Beta (95% confidence interval) and *p*-values for univariate regression analyses. *CI* confidence interval, *CIMT* carotid intima-media thickness, *eGFR* estimated glomerular filtration rate, *GDF-15 *growth differentiation factor 15, *HDL* high-density lipoprotein, *HDP* hypertensive disorder of pregnancy, *hs-CRP *high sensitivity C-reactive protein, *Ln* natural logarithm, *NT-proBNP* N-terminal pro-brain natriuretic peptide, *PWV* pulse wave velocity, *RWT* relative wall thickness, *SAA1* serum amyloid A1, s*HLA-G* soluble human leukocyte antigen-G, *TVR* total vascular resistance

‡ Indicates negative beta coefficients that, due to rounding appeared as negative zero values. These are reported as zero in the table

**Table 5:** Unadjusted regression analyses for the prior PE group (*n* = 132). Associations between circulating biomarkers and cardiovascular structure and function at follow-up. Significant associations (*p* < 0.05) are marked with asterix (*). n denotes number of women included in each investigation, by study group

Circulating biomarkers	PWV (m/s)		CIMT (mm)		RWT		TVR (dyne/s/cm^− 5^)		E/e´ ratio	
	b (95% CI)	*p*	b (95% CI)	*p*	b (95% CI)	*p*	b (95% CI)	*p*	b (95% CI)	*p*
Non-HDL cholesterol (mmol/L)	0.35 (0.12–0.58)	< 0.01*	0.03 (0.01–0.05)	< 0.01*	-0.01 (-0.03–0.00)	0.14	42.27 (-47.56–132.11)	0.35	-0.02 (-0.50–0.46)	0.92
n	116		122		54		51		54	
eGFR (ml/min/1.73 m²)	-0.01 (-0.03–0.00)	0.11	0.00 ‡ (-0.00–0.00)	0.87	0.00 (-0.00–0.00)	0.41	0.56 (-5.45–6.58)	0.85	-0.01 (-0.04–0.02)	0.35
n	115		121		54		51		54	
Ln glucose (mmol/L)	2.79 (0.53–5.06)	0.02*	0.06 (-0.11–0.24)	0.48	0.01 (-0.12–0.15)	0.86	373.83 (-340.55–1088.22)	0.30	0.52 (-3.28–4.32)	0.79
n	113		120		54		51		54	
Ln hs-CRP (mg/L)	0.38 (0.13–0.64)	< 0.01*	0.02 (-0.00–0.04)	0.10	0.01 (-0.00–0.03)	0.144	-38.11 (-140.18–63.76)	0.455	0.10 (-0.43–0.64)	0.70
n	116		122		54		51		54	
Ln sHLA-G (µmol/L)	0.09 (-0.17–0.36)	0.47	0.01 (-0.01–0.03)	0.48	-0.01 (-0.03–0.01)	0.26	11.95 (-99.86–123.76)	0.83	0.07 (-0.49–0.64)	0.80
n	108		121		53		50		53	
Ln SAA1 (ng/ml)	0.12 (-0.08–0.32)	0.23	0.00 ‡ (-0.02–0.02)	1.00	0.00 ‡ (-0.02–0.01)	0.78	23.34 (-59.67–106.36)	0.57	-0.06 (-0.50–0.37)	0.770
n	115		121		53		50		53	
Ln GDF-15 (ng/L)	-0.40 (-1.31–0.52)	0.39	-0.04 (-0.11–0.04)	0.33	-0.04 (-0.09–0.02)	0.21	-241.46 (-524.41–41.49)	0.09	-0.23 (-1.91–1.45)	0.78
n	109		121		53		50		53	
Ln NT-proBNP (ng/L)	0.07 (-0.21–0.35)	0.62	0.00 ‡ (-0.02–0.02)	0.86	0.00 ‡ (-0.02–0.02)	0.94	-27.32 (-135.98–81.34)	0.62	0.05 (-0.52–0.62)	0.86
n	110		122		54		51		54	

*b* Beta (95% confidence interval) and *p*-values for univariate regression analyses. *CI* confidence interval, *CIMT* carotid intima-media thickness, *eGFR* estimated glomerular filtration rate, *GDF-15 *growth differentiation factor 15, *HDL *high-density lipoprotein, *hs-CRP *high sensitivity C-reactive protein, *Ln *natural logarithm, *NT-proBNP* N-terminal pro-brain natriuretic peptide, *PE* preeclampsia, *PWV* pulse wave velocity, *RWT* relative wall thickness, *SAA1 *serum amyloid A1, *sHLA-G* soluble human leukocyte antigen-G, *TVR *total vascular resistance

‡ Indicates negative beta coefficients that, due to rounding appeared as negative zero values. These are reported as zero in the table

In univariate regression analyses for the control group, non-HDL cholesterol was significantly associated with CIMT and E/e´ratio. Additionally, NT-proBNP was also significantly negatively associated with relative wall thickness (RWT) (Supplementary Table S6).

Median cTnT concentrations were similar in all postpartum study groups (Table 2). Furthermore, when participants were stratified by cTnT (≤3 ng/L and > 3 ng/L), median values of the cardiovascular assessment variables showed no significant differences between any study group (Supplementary Tables S7-S10).

### Elevated Postpartum Blood Pressure and Other Risk Factors for CVD

The 2024 European Society for Cardiology (ESC) Guidelines on the management of elevated blood pressure and hypertension [37] emphasize the benefit of a blood pressure lower than the traditional hypertension definition (an office blood pressure ≥ 140 mmHg systolic and/or ≥ 90 mmHg diastolic). We therefore assessed if the previous HDP group (*n* = 176) differed in modifiable risk factors and investigated biomarkers at follow-up, when dichotomized (at one or three years follow-up) into normotensive (*n* = 118; normal blood pressure: SBP < 120 mmHg and DBP < 80 mmHg) or not (*n* = 58; elevated blood pressure, stage 1 or stage 2 hypertension: SBP ≥120 and DBP ≥80 mmHg, as well as women using antihypertensive medication). As shown in Supplementary Table S11; these groups within the prior HDP group differed statistically significantly in median levels of BMI, overweight rates, LDL cholesterol, non-HDL cholesterol, triglycerides, eGFR, glucose, hs-CRP, PWV, CIMT and TVR. Even though not statistically significant, the prior HDP group that was not normotensive at follow-up (*n* = 58), additionally showed a strong trend towards more adverse kidney function, with higher creatinine level and higher rates of low eGFR (< 90 ml/min/1.73 m^2^) and elevated albumin/creatinine ratio (≥3 mg/mmol), compared to the normotensive group (*n* = 118). In summary, albeit not all group differences were statistically significant, the normotensive subgroup within the previous HDP group had a lower total burden of modifiable cardiovascular risk factors than the group with elevated blood pressure levels or hypertension (Supplementary Table S11).

As women with stage 2 hypertension normally would receive follow-up, we also conducted an analysis comparing the group with elevated or stage 1 hypertensive BP (*n* = 49; SBP ≥120 mmHg and < 140 mmHg and DBP ≥80 mmHg and < 90 mmHg) to the normotensive group (*n* = 118; SBP < 120 mmHg and DBP < 80 mmHg), finding similar group differences (data not shown). The same was true when the normotensives were grouped together with the elevated blood pressure group (*n* = 142; SBP < 130 mmHg and DBP < 80 mmHg) and compared with stage 1 and stage 2 hypertension (*n* = 34; SBP ≥130 mmHg and/or DBP ≥ 80 mmHg), expect from no significant differences in eGFR and glucose levels (data not shown).

## Discussion

In our present study, non-HDL cholesterol, glucose and hs-CRP, were significantly associated with markers of arterial structure and function (PWV and CIMT) one or three years following HDP. The prior HDP group also showed more unfavorable cardiometabolic profiles compared to controls, including higher PWV and TVR levels, more frequent high blood pressures and metabolic syndrome, as well as non-significantly more frequent overweight and obesity. However, none of the circulating biomarkers (hs-CRP, non-HDL cholesterol, serum glucose, eGFR, cTnT, NT-proBNP, GDF-15. sHLA-G, SAA1) differed in median values between women with prior HDP and controls, except for urine albumin/creatinine ratio.

In our cohort, women with prior HDP did not differ significantly from controls in lipid values. Several follow-up studies have, however, shown significantly worse lipid profiles after HDP, compared with normotensive pregnancies [22, 38]. Our lack of statistically significant differences may reflect lower statistical power, due to smaller sample sizes, as well as a generally healthier postpartum HDP population.

In our univariate regression analyses, non-HDL cholesterol was significantly associated with increased PWV in the prior HDP and PE groups. Few studies have examined links between circulating lipids and PWV in women after HDP. More broadly, research on these associations in young populations is limited. Our results align with a Finnish cohort (male and female, age 30–45 years) that found associations between non-HDL cholesterol and increased arterial stiffness [39], and with a Chinese cohort identifying non-HDL cholesterol as a potential risk factor for arterial stiffness, particularly in younger individuals [40]. Arterial stiffness increases with blood pressure [41], which may partly mediate the association between non-HDL cholesterol and PWV observed in our prior HDP and PE groups. Persistent vascular dysfunction following HDP may further explain the association in this subgroup [10].

Glucose and hs-CRP were also significantly associated with PWV at follow-up in our prior HDP and PE groups. As with non-HDL cholesterol, few studies have examined associations between inflammatory or metabolic biomarkers and arterial stiffness following HDP. Nonetheless, our findings are consistent with previous work in diverse general populations across sex and age groups, that reported significant associations between glucose and hs-CRP and increased PWV [41–44]. These associations in our and other studies support the impact of inflammation on arterial stiffness and CVD development [15]. Less favorable cardiometabolic profiles, including higher rates of metabolic syndrome and hypertension in our prior HDP and PE groups, may influence both inflammation and arterial stiffness and therefore partly explain these associations. Of note, a recent paper found that hs-CRP independently predicts cardiovascular events in the general population (without a history of atherosclerotic CVD), and ranks above several traditional CVD risk factors, except for age, sex, smoking and SBP, which further supports the use of hs-CRP in CVD risk assessment among women after HDP [45].

Non-HDL cholesterol showed a significant association with CIMT across all our study groups, except for in the small prior GH group. This finding is consistent with previous studies showing an association between non-HDL cholesterol and CIMT in both younger and older healthy populations [46, 47]. Although median CIMT did not differ between groups overall, except for significantly higher CIMT in the prior GH group, other studies report higher levels of CIMT following HDP [10]. Given the benefits of reducing modifiable CVD risk factors in the general population [48], women with a history of recent HDP warrant particular attention because of their increased risk of premature CVD and higher prevalence of other CVD risk factors [1, 5, 49].

NT-proBNP is a biomarker of cardiomyocyte stretching and is frequently used in clinical practice to diagnose heart failure [17]. We have previously shown elevated levels of NT-proBNP during HDP pregnancies and associations between NT-proBNP and surrogate markers of placental dysfunction (sFlt-1/PlGF ratio and birthweight percentile) in a similar cohort [27]. Similarly to our previous report of a larger patient group of our HAPPY cohort [27], no differences in postpartum NT-proBNP were observed between study groups in the present smaller study. Also, for the prior HDP groups, NT-proBNP showed no significant associations with any cardiovascular assessment variables at follow-up. However, for the control group, NT-proBNP was significantly associated with lower RWT. In general, NT-proBNP levels above age group reference values are not uncommon in females without classical CVD risk factors [50], which may explain this potentially spurious finding together with the relatively small sample size. The smoking rate was in general low in our cohort, limiting subanalyses, but more women in the control group with available echocardiography examinations were smokers compared to the previous HDP group (*n* = 7 of 34 vs. *n* = 0 of 60, respectively) [13]. Smoking is associated with more adverse cardiac structure and function, as well as increased NT-proBNP [50, 51], and may therefore partly explain this association in the control group of NT-proBNP being associated with lower RWT. In support of a potential effect of smoking, a sensitivity analysis in our control cohort, excluding current smokers, showed no significant association between NT-proBNP and RWT (data not shown).

At one- or three-year follow-up, urine albumin/creatinine ratio was significantly higher in our prior HDP, PE and GH groups, compared to controls, while serum creatinine and eGFR did not significantly differ between groups. Nevertheless, albumin/creatinine ratio values above reference range were more common in women with prior HDP (as well as PE and GH subgroups), compared to controls. As for the two women with abnormal eGFR (< 90 ml/min/1.73 m²) and albumin/creatinine ratio (≥3 mg/mmol) at the postpartum follow-up, they were both women with PE in their index pregnancy. These biomarkers may reflect reduced kidney function, but measurements were only taken once, and definitive conclusions cannot be made. However, both women had no known kidney disease prior to examination, but they had elevated levels of several other CVD risk factors, including overweight (both prior to and during pregnancy and postpartum), elevated blood pressure or stage 1 hypertension, and one was taking antihypertensive medications at time of follow-up. Although eGFR was not significantly associated with any cardiovascular assessment variables in our study, the observed alterations in kidney markers are in line with previous reports showing an increased long-term risk of chronic kidney disease (CKD) and end-stage renal failure following HDP [25, 52]. Accordingly, some experts recommend considering renal follow-up after HDP to detect possible undiagnosed CKD and improve long-term renal health [52].

We found less favorable cardiometabolic and CVD risk profiles in women with prior HDP compared to controls. This includes higher levels of modifiable CVD risk factors, such as higher BMI and blood pressure levels and more frequent metabolic syndrome and overweight. Because established CVD risk evaluation tools are not suitable for young women following HDP [28], the most effective follow-up strategy to identify women with the highest CVD risk postpartum remains unknown. We have previously shown that echocardiography adds limited information beyond well-known risk factors in CVD risk evaluation in clinically healthy women following HDP [13]. Our findings from this current study underscores the importance of CVD follow-up after HDP, in accordance with national and international obstetric and cardiovascular guidelines [14, 53–57].

The ACC/AHA blood pressure categories used in this paper have been criticized, especially in older patients, because of the lack of evidence for further CVD risk reduction by blood pressure reduction from 130–139/80–89 mmHg in untreated patients without previous cardiovascular events [58]. However, CVD risk is a continuum, as are CVD risk factors, including blood pressure levels. A mean SBP < 130 mmHg is associated with fewer major cardiovascular events than SBP ≥ 130 mmHg [59]. As presented in the results section, all combinations of blood pressure categories showed elevated levels of the investigated CVD risk factors in the higher blood pressure groups, whether the “normal” dichotomy was set at 120 or 130 mmHg. Our finding of a lower total burden of cardiovascular risk factors at follow-up in the normotensive subgroup within the previous HDP group (across all thresholds investigated for “normal” BP) supports a focus on good blood pressure control already from the early postpartum years.

### Strengths and Limitations

Our study cohort is phenotypically well-defined and thoroughly examined with comprehensive clinical data and detailed circulating biomarker and cardiovascular assessment data, which strengthens our study. Additionally, we have used validated pregnancy complication definitions and included an age-matched control group. To evaluate the effects of HDP more specifically, women with chronic diseases that may elevate CVD risk (e.g. diabetes) were excluded. For the same reason, no woman with prior preeclampsia and/or GDM was included as a control. Although circulating lipids, glucose and PWV could be influenced by recent food intake, excluding the three participants who did not meet our strict fasting criteria had no effect on the findings.

Our cardiovascular assessment examinations are strengthened by few performing examiners and the use of the same apparatus for all participants, to ensure uniform conditions for examination and analyses of results. All echocardiographic examinations were conducted by one of two experienced cardiologists, who were unaware of each study participants´ pregnancy outcome during examination. PWV and CIMT were examined by one trained examiner, with “excellent” intraclass correlation coefficients [12].

Our study is limited by variations in available cardiovascular assessments for the included study participants, mainly caused by limited availability of the echocardiographic examinations. This may introduce selection bias, as well as difficulties comparing the associations between circulating lipids and different cardiovascular assessments, due to reduced statistical power for the echocardiographic variables. Regression analyses for the echocardiographic variables for the GH group were not conducted due to small sample size (*n* = 6). We did not adjust for potential confounders because the primary aim of the present analyses was to estimate the associations between circulating biomarkers and cardiovascular structure and function 1–3 years postpartum. Accordingly, these analyses are intended as an initial step towards potential clinical use, rather than investigating underlying pathophysiological mechanisms. Nonetheless, well-known CVD risk factors (e.g., high age, overweight and hypertension) may influence associations and should always be included in an overall clinical evaluation of CVD risk. Also, the impact of primary prevention strategies, including increasing physical activity, was not assessed in this study, and warrants further investigation.

Our study cohort was demographically relatively homogenous, including clinically healthy women of young (premenopausal) age, mostly of white ethnicity with high education. Representative of the general Norwegian population, these women live in a high-income country with free-of charge antenatal and obstetric health care, which may improve both pregnancy and general health outcomes, but external validity to more heterogenous populations with different access to healthcare may be limited. Also, bias may be introduced by the inclusion of both one- and three-year follow-up data, but main conclusions remained the same following sensitivity analyses for one-year data.

## Conclusion

Circulating CVD biomarkers were associated with markers of arterial stiffness following HDP. Non-HDL cholesterol, glucose and hs-CRP were significantly associated with PWV one or three years following HDP and PE. In addition, women with prior HDP, PE and normotensive pregnancies, showed significant associations between non-HDL cholesterol and CIMT at follow-up. Women with prior HDP and PE displayed more adverse cardiometabolic profiles, including more adverse blood pressure levels, higher PWV and TVR and more frequent metabolic syndrome, compared to controls. Circulating biomarkers showed no significant associations with echocardiographic measures one or three years following HDP.

Our findings support previous reports of associations between metabolic and inflammatory biomarkers and increased arterial stiffness. Circulating biomarkers are easily measured and more widely accessible across diverse socioeconomic settings than more resource-demanding cardiovascular assessments. Such biomarkers could potentially enable improved CVD risk assessment and personalized follow-up following HDP when combined with other CVD risk factors, such as blood pressure and BMI. Our findings underscore the clinical importance of identifying and addressing modifiable risk factors for CVD after HDP, to enable CVD prevention from subclinical stages before disease progression becomes manifest. Our study supports that screening for hypertension, dyslipidemia, hyperglycemia and overweight should be prioritized after HDP to enable lifestyle interventions and risk reduction. In addition, hs-CRP may be considered a potential add-on biomarker for CVD assessment in the future.

## Supplementary Information

Below is the link to the electronic supplementary material.

Supplementary Material 1 (DOCX 106 KB)

## Data Availability

Due to ongoing clinical follow-up, the sensitive nature of the study data material and the patient informed consents (including ethical body approval) access to data material is restricted. Requests for access to portions of the anonymized dataset may be directed to the corresponding author.
