# Supplementary material for: Circulating Cardiovascular Biomarkers 1–3 Years Following Hypertensive Pregnancy Disorders: Associations With Postpartum Cardiovascular Structure and Function
Source: Reprod Sci. 2026 Aug 21;33(9):1659–73. doi: 10.1007/s43032-026-02171-y (PMC13562120; doi:10.1007/s43032-026-02171-y)
Supplement: Supplementary file 1 [file 43032_2026_2171_MOESM1_ESM.docx]

**Supplementary Figure S1.** Flowchart of the inclusion and exclusion to this HAPPY sub-study (health after pregnancy complications) one or three years postpartum.

Singleton delivery at Oslo University Hospital - Ullevål
1 or 3 years prior (2013 – 2019)

Index pregnancy with HDP, GDM or uncomplicated pregnancy ***or*** included in previous study in index pregnancy

Participants approached by the study investigators

Patients declining
Multiple delivery in index pregnancy

n=449

Included in the HAPPY study 1or 3 years postpartum (2014-2020)

n=20

Cardiovascular assessment or circulating biomarkers unavailable

n=429

Cardiovascular assessment and circulating biomarkers available

n=154

Breastfeeding

Pregnant

Pregnant after index pregnancy

Egg donation

Intercurrent disease

Chronic hypertension without PE

DM or GDM

Delivery of infant with chromosomal abnormalities

Controls excluded if:

Fetal growth restricted baby

Previous PE or preterm birth

n=275

Included to the HAPPY sub-study (210 at 1 year and 65 at 3 years postpartum)

PE (n=132), GH (n=44) or normal pregnancy (n=99)

DM: diabetes mellitus; GDM: gestational diabetes mellitus; GH: gestational hypertension; HDP: hypertensive disorders of pregnancy (including both PE and GH patients); PE: preeclampsia

**Supplemental Table S1.** Computed echocardiographic variables (previously published by us [1].

| Echocardiographic measurements | Based on variables | Formula |
| --- | --- | --- |
| TVR (dyne/s/cm^-5^) [2] | - LVCODopp (L/min) - MAP (mmHg) | 80 x(MAP/LVCODopp) |
| LVM (g) [3] | - LVIDd (cm) - SWd (cm) - LVPWd (cm) | Devereux formula:  0.8*(1.04(((LVIDd + SWd + LVPWd)^3^) - (LVIDd^3^))) + 0.6 |
| RWT [4] | - LVPWd (cm) - LVIDd (cm) | 2*LVPWd/LVIDd |

TVR: total vascular resistance; LVCODopp: left ventricular cardiac output imaged by Doppler technique; MAP: mean arterial blood pressure; LVM: left ventricular mass; LV: left ventricular; LVIDd: LV inner dimension in diastole; LVPWd: LV posterior wall diameter in diastole; SWd: septal wall diameter in diastole; RWT: relative wall thickness.

**Supplementary Table S2.** Normal values for echocardiographic variables (previously published by us [1].

|  | Normal range | | |
| --- | --- | --- | --- |
| VASCULAR STRUCTURE AND FUNCTION | | | |
| ^#^PWV (m/s) [5] | 5.2 – 8.0 | | |
| CIMT (mm) [6] | **≤**0.9 | | |
| CARDIAC MORPHOLOGY/STRUCTURE | | | |
| ^§^ SWd (cm) [7] | 0.6-0.9 | | |
| ^§^LVM (g) [7] | 66-150 | | |
| ^§^RWT [7] | 0.22-0.42 | | |
| CARDIAC FUNCTION | |  |  |
| TVR (dyne/s/cm^-5^) [2] | 900-1440 | | |
| ^#§^SV (mL) [8] | 47.5-75.5 | | |
| ^§^CO (L/minute) [8] | 3.3– 5.3 | | |
| ^§^LVEF (%) [7] | 54-74 | | |
| DIASTOLIC FUNCTION | |  |  |
| ^#^ E (cm/s) [9] | 64-96 | | |
| ^#^A (cm/s) [9] | 33-63 | | |
| E/A ratio [10] | >0.8 | | |
| E/e´ ratio [10] | <8 | | |

TVR: total vascular resistance; SV: stroke volume by Doppler technique; CIMT: carotid intima-media thickness; CO: cardiac output; LVEF: left ventricular ejection fraction (biplane); LVM: left ventricular mass; PWV: pulse wave velocity; RWT: relative wall thickness; SWd: septal wall diameter in diastole.

^#^Age-specific normal range for age <40 years. ^§^Female-specific reference values.

**Supplementary Table S3.** Pregnancy characteristics of the study population (n=275), by index pregnancy group. Data are presented as medians (interquartile range) for continuous variables. Categorical variables are presented as rates (%). A p-value <0.05 is marked with asterix (*).

| Pregnancy characteristics | Controls  (n=99) | HDP  (n=176) | p-value | PE  (n=132) | p-value | GH  (n=44) | p-value |
| --- | --- | --- | --- | --- | --- | --- | --- |
| Age at delivery (years) | 33 (31 - 36) | 33 (30 - 37) | 0.88 | 33(30 - 37) | 0.94 | 33 (32 - 36) | 0.57 |
| Weight prepregnancy (kg) | 62 (58 - 70) | 65 (58 - 74) | 0.18 | 64 (58 - 74) | 0.41 | 67 (61 - 74) | 0.06 |
| Height (cm) | 168 (164 - 172) | 167 (163 - 171) | 0.33 | 167 (163 - 171) | 0.13 | 168 (165 - 173) | 0.53 |
| BMI prepregnancy (kg/m²) | 22.4 (20.9 - 24.7) | 23.5 (21.3 - 25.7) | 0.03* | 23.4 (21.3 - 25.9) | 0.05 | 23.6 (21.5 - 25.5) | 0.06 |
| Overweight (BMI ≥ 25 kg/m² and BMI <30 kg/m²) | 19.2% (19) | 23.9% (42) | 0.33 | 24.2% (32) | 0.30 | 22.7% (10) | 0.65 |
| Obesity (BMI ≥ 30 kg/m²) | 4.0% (4) | 7.4% (13) | 0.25 | 6.8% (9) | 0.34 | 9.1% (4) | 0.25 |
| Birthweight (g) | 3490 (3257 - 3745) | 2836 (2104 - 3464) | <0.01* | 2597 (1838 - 3250) | <0.01* | 3471 (3054 - 3721) | 0.35 |
| FGR (< 3rd percentile) | 0.0% (0) | 29.0% (51) | <0.01* | 38.6% (51) | <0.01* | 0.0% (0) | - |
| Female offspring | 54.5% (54) | 49.4% (87) | 0.42 | 50.0% (66) | 0.49 | 47.7% (21) | 0.45 |
| Gestational age at delivery (weeks) | 39.3 (38.9 - 39.9) | 38.0 (35.6 - 39.7) | <0.01* | 37.3 (34.3 – 39.0) | <0.01* | 39.7 (38.6 - 40.6) | 0.53 |
| Primiparous | 45.5% (45) | 68.2% (120) | <0.01* | 71.2% (94) | <0.01* | 59.1% (26) | 0.13 |
| Systolic BP <week 20 (mmHg) | 110 (100 - 116) | 116 (110 - 123) | <0.01* | 115 (108 - 122) | <0.01* | 120 (110 - 125) | <0.01* |
| Diastolic BP <week 20 (mmHg) | 66 (60 - 72) | 73 (68 - 80) | <0.01* | 72 (68 - 80) | <0.01* | 78 (70 - 82) | <0.01* |
| MAP <week 20 (mmHg) | 80 (76 - 87) | 88 (83 - 93) | <0.01* | 86 (82 - 93) | <0.01* | 92 (84 - 96) | <0.01* |

The Mann-Whitney U test for continuous variables and Chi square or Fisher´s mid-p corrected test for categorical variables comparing pregnancy complication groups (e.g. HDP) to controls. Values are presented as medians (ranges) or rates (n). BMI: body mass index; BP: blood pressure; GH: gestational hypertension; HDP: hypertensive disorders of pregnancy (including both PE and GH patients); MAP: mean arterial blood pressure; PE: preeclampsia.

**Supplementary Table S4.** Kidney function markers outside reference values (n=275), by index pregnancy group. A p-value <0.05 is marked with asterix (*).

| Kidney function markers  outside reference range | Controls (n=99)  % (n) | HDP (n=176)  % (n) | p-value | PE (n=132) % (n) | p-value | GH (n=44)  % (n) | p-value |
| --- | --- | --- | --- | --- | --- | --- | --- |
| eGFR (ml/min/1.73 m²)  (60-89 ml/min/1.73 m²) | 4.0% (4) | 7.4% (13) | 0.27 | 9.1% (12) | 0.14 | 2.3% (1) | 1.00 |
|  |  |  |  |  |  |  |  |
| Albumin/creatinine ratio  (≥ 3 mg/mmol) | 2.0% (2) | 9.7% (17) | 0.02* | 8.3% (11) | 0.04* | 13.6% (6) | 0.01* |

The chi-square or Fisher´s mid-p corrected test comparing pregnancy complication groups (e.g. HDP) to controls. Values are presented as rates (n). eGFR: estimated glomerular filtration rate.

**Supplementary Table S5.** Associations between circulating biomarkers and vascular structure and function at 1–3-year follow-up for the GH group (n=44). Significant associations (p<0.05) are marked with asterix (*).

| **Circulating biomarkers** | **PWV (m/s)** | | **CIMT (mm)** | |
| --- | --- | --- | --- | --- |
|  | **b (95% CI)** | **p** | **b (95% CI)** | **p** |
| **Non-HDL cholesterol (mmol/L)** | 0.32  (-0.07 - 0.72) | 0.11 | 0.03  (-0.00 - 0.06) | 0.05 |
| **n** | 38 |  | 40 |  |
| **eGFR (ml/min/1.73 m²)** | -0.01  (-0.04 - 0.02) | 0.53 | 0.00  (-0.00 - 0.00) | 0.14 |
| **n** | 38 |  | 40 |  |
| **Ln glucose (mmol/L)** | 5.73  (1.76 - 9.69) | <0.01* | 0.17  (-0.17 - 0.50) | 0.31 |
| **n** | 37 |  | 38 |  |
| **Ln hs-CRP (mg/L)** | 0.36  (-0.04 - 0.76) | 0.08 | -0.02  (-0.07 - 0.02) | 0.25 |
| **n** | 38 |  | 39 |  |
| **Ln sHLA-G (µmol/L)** | 0.40  (-0.31 - 1.11) | 0.26 | 0.02  (-0.04 - 0.08) | 0.44 |
| **n** | 36 |  | 40 |  |
| **Ln SAA1 (ng/ml)** | 0.31  (-0.03 - 0.66) | 0.08 | -0.01  (-0.05 - 0.03) | 0.76 |
| **n** | 38 |  | 40 |  |
| **Ln GDF-15 (ng/L)** | -0.30  (-1.91 - 1.32) | 0.71 | -0.06  (-0.18 - 0.05) | 0.24 |
| **n** | 37 |  | 40 |  |
| **Ln NT-proBNP (ng/L)** | 0.23  (-0.27 - 0.74) | 0.35 | 0.02  (-0.02 - 0.07) | 0.23 |
| **n** | 37 |  | 40 |  |

b: Beta (95% confidence interval) and p-values for univariate regression analyses. CI: confidence interval; CIMT: carotid intima-media thickness; eGFR: estimated glomerular filtration rate; GDF-15: growth differentiation factor 15; GH: gestational hypertension; HDL: high-density lipoprotein; hs-CRP: high sensitivity C-reactive protein; Ln: natural logarithm; NT-proBNP: N-terminal pro-brain natriuretic peptide; PE: preeclampsia; PWV: pulse wave velocity; SAA1: serum amyloid A1; sHLA-G: soluble human leukocyte antigen-G.

**Supplementary Table S6.** Associations between circulating biomarkers and cardiovascular structure and function at 1–3-year postpartum follow-up for the control group (n=99). Significant associations (p<0.05) are marked with asterix (*).

| **Circulating biomarkers** | **PWV (m/s)** | | **CIMT (mm)** | | **RWT** | | **TVR (dyne/s/cm^-5^)** | | **E/e´ ratio** | |
| --- | --- | --- | --- | --- | --- | --- | --- | --- | --- | --- |
|  | **b (95% CI)** | **p** | **b (95% CI)** | **p** | **B (95% CI)** | **p** | **b (95% CI)** | **p** | **b (95% CI)** | **p** |
| **Non-HDL cholesterol  (mmol/L)** | 0.22  (-0.00 - 0.44) | 0.05 | 0 (0.01 - 0.41) | 0.01* | 0.01  (-0.02 - 0.03) | 0.56 | 122.14  (-23.25 - 267.54) | 0.10 | 0.87  (0.26 - 1.48) | 0.01* |
| **n** | 89 |  | 94 |  | 34 |  | 31 |  | 33 |  |
| **eGFR  (ml/min/1.73 m²)** | -0.01  (-0.02 - 0.01) | 0.40 | 0.00 ‡  (-0.00 - 0.00) | 0.88 | 0.00  (-0.00 - 0.00) | 0.69 | 3.62  (-4.89 - 12.12) | 0.39 | -0.04  (-0.07 - 0.00) | 0.06 |
| **n** | 88 |  | 93 |  | 33 |  | 30 |  | 32 |  |
| **Ln glucose  (mmol/L)** | 1.92  (-0.28 - 4.13) | 0.09 | -0.01  (-0.20 - 0.19) | 0.92 | 0.00 ‡ (-0.25 - 0.25) | 0.99 | 1350.30  (-24.38 - 2724.98) | 0.05 | -3.27  (-9.96 - 3.42) | 0.33 |
| **n** | 89 |  | 94 |  | 34 |  | 31 |  | 33 |  |
| **Ln hs-CRP (mg/L)** | 0.15  (-0.10 - 0.39) | 0.24 | 0.01  (-0.01 - 0.02) | 0.53 | 0.00 ‡ (-0.03 - 0.03) | 0.87 | -50.98  (-208.41 - 106.45) | 0.51 | 0.59  (-0.11 - 1.30) | 0.10 |
| **n** | 89 |  | 94 |  | 34 |  | 31 |  | 33 |  |
| **Ln sHLA-G (µmol/L)** | -0.02  (-0.28 - 0.23) | 0.86 | -0.01  (-0.03 - 0.01) | 0.18 | -0.01 (-0.03 - 0.02) | 0.64 | 88.86  (-45.81 - 223.53) | 0.19 | 0.12  (-0.50 - 0.74) | 0.70 |
| **n** | 84 |  | 93 |  | 33 |  | 30 |  | 32 |  |
| **Ln SAA1 (ng/ml)** | 0.03  (-0.16 - 0.22) | 0.74 | 0.00 ‡  (-0.02 - 0.01) | 0.56 | 0.00 ‡ (-0.02 - 0.02) | 0.99 | -53.59  (-152.83 - 45.65) | 0.28 | 0.46  (-0.00 - 0.92) | 0.05 |
| **n** | 88 |  | 93 |  | 33 |  | 30 |  | 32 |  |
| **Ln GDF-15 (ng/L)** | 0.80  (-0.09 - 1.69) | 0.08 | -0.02  (-0.09 - 0.05) | 0.62 | 0.01  (-0.09 - 0.10) | 0.90 | 41.97  (-508.29 - 592.22) | 0.88 | -0.64  (-3.23 - 1.94) | 0.61 |
| **n** | 87 |  | 94 |  | 34 |  | 31 |  | 33 |  |
| **Ln NT-proBNP (ng/L)** | -0.17  (-0.43 - 0.09) | 0.19 | -0.01  (-0.03 - 0.01) | 0.24 | -0.02  (-0.05 - -0.00) | 0.03* | -26.18  (-160.79 - 108.44) | 0.69 | 0.61  (-0.01 - 1.23) | 0.05 |
| **n** | 87 |  | 94 |  | 34 |  | 31 |  | 33 |  |

b: Beta (95% confidence interval) and p-values for univariate regression analyses. CI: confidence interval; CIMT: carotid intima-media thickness; eGFR: estimated glomerular filtration rate; GDF-15: growth differentiation factor 15; HDL: high density lipoprotein; hs-CRP: high sensitivity C-reactive protein; Ln: natural logarithm; NT-proBNP: N-terminal pro brain natriuretic peptide; PWV: pulse wave velocity; RWT: relative wall thickness; SAA1: serum amyloid A1; sHLA-G: soluble human leukocyte antigen-G; TVR: total vascular resistance.
‡ Indicates negative beta coefficients that, due to rounding appeared as negative zero values. These are reported as zero in the table.

**Supplementary Table S7.** Median (IQR) levels of cardiovascular assessment parameters in participants stratified by cardiac troponin T (cTnT) concentration (≤3 ng/L vs. >3 ng/L) for the HDP group (n=176) at 1-3-year postpartum follow-up. Group differences were evaluated using the Mann–Whitney U test.

|  | PWV (m/s) | CIMT (mm) | RWT | TVR (dyne/s/cm^-5^) | E/e´ ratio |
| --- | --- | --- | --- | --- | --- |
|  | Median (IQR) | Median (IQR) | Median (IQR) | Median (IQR) | Median (IQR) |
| cTnT (ng/L) ≤3 | 6.2  (5.7 - 6.9) | 0.48  (0.43 - 0.52) | 0.28  (0.25 - 0.32) | 1524.96  (1402.68 - 1726.44) | 6.23  (5.89 - 7.65) |
| n | 132 | 143 | 56 | 52 | 55 |
| cTnT (ng/L) >3 | 6.2  (5.8 - 6.6) | 0.46  (0.41 - 0.48) | 0.27  (0.25 – 0.30) | 1325.49  (1301.59 - 1561.56) | 5.66  (5.27 - 6.56) |
| n | 22 | 19 | 4 | 4 | 4 |
| p-value | 0.59 | 0.28 | 0.60 | 0.31 | 0.21 |

CIMT: carotid intima-media thickness; cTnT: cardiac troponin T; HDP: hypertensive disorders of pregnancy (including both PE and GH patients); IQR: interquartile range; PWV: pulse wave velocity; RWT: relative wall thickness; TVR: total vascular resistance.

**Supplementary Table S8.** Median (IQR) levels of cardiovascular assessment parameters in participants stratified by cardiac troponin T (cTnT) concentration (≤3 ng/L vs. >3 ng/L) for the PE group (n=132) at 1-3-year postpartum follow-up. Group differences were evaluated using the Mann–Whitney U test.

|  | PWV (m/s) | CIMT (mm) | RWT | TVR (dyne/s/cm^-5^) | E/e´ ratio |
| --- | --- | --- | --- | --- | --- |
|  | Median (IQR) | Median (IQR) | Median (IQR) | Median (IQR) | Median (IQR) |
| cTnT (ng/L) ≤3 | 6.2  (5.7 - 7.0) | 0.47  (0.42 - 0.52) | 0.29  (0.26 - 0.33) | 1515.04  (1380.53 - 1696.69) | 6.22  (5.89 - 7.65) |
| n | 100 | 109 | 50 | 47 | 50 |
| cTnT (ng/L) >3 | 6.4  (5.5 - 6.8) | 0.45  (0.44 - 0.48) | 0.27  (0.25 – 0.30) | 1325.49  (1301.59 - 1561.56) | 5.66  (5.27 - 6.56) |
| n | 16 | 13 | 4 | 4 | 4 |
| p-value | 0.81 | 0.80 | 0.50 | 0.31 | 0.22 |

CIMT: carotid intima-media thickness; cTnT: cardiac troponin T; IQR: interquartile range; PE: preeclampsia; PWV: pulse wave velocity; RWT: relative wall thickness; TVR: total vascular resistance.

**Supplementary Table S9.** Median (IQR) levels of cardiovascular assessment parameters in participants stratified by cardiac troponin T (cTnT) concentration (≤3 ng/L vs. >3 ng/L) for the GH group (n=44) at 1-3-year postpartum follow-up. Group differences were evaluated using the Mann–Whitney U test.

|  | PWV (m/s) | CIMT (mm) |
| --- | --- | --- |
|  | Median (IQR) | Median (IQR) |
| cTnT (ng/L) ≤3 | 6.2  (5.8 - 6.8) | 0.49  (0.46 - 0.53) |
| n | 32 | 34 |
| cTnT (ng/L) >3 | 6.0  (5.9 - 6.4) | 0.44  (0.38 - 0.54) |
| n | 6 | 6 |
| p-value | 0.43 | 0.18 |

CIMT: carotid intima-media thickness; cTnT: cardiac troponin T; GH: gestational hypertension; IQR: interquartile range; PWV: pulse wave velocity; RWT: relative wall thickness; TVR: total vascular resistance.

**Supplementary Table S10.** Median (IQR) levels of cardiovascular assessment parameters in participants stratified by cardiac troponin T (cTnT) concentration (≤3 ng/L vs. >3 ng/L) for the control group (n=99) at 1-3-year postpartum follow-up. Group differences were evaluated using the Mann–Whitney U test.

|  | PWV (m/s) | CIMT (mm) | RWT | TVR (dyne/s/cm^-5^) | E/e´ ratio |
| --- | --- | --- | --- | --- | --- |
|  | Median (IQR) | Median (IQR) | Median (IQR) | Median (IQR) | Median (IQR) |
| cTnT (ng/L) ≤3 | 5.9  (5.5 - 6.4) | 0.45  (0.43 - 0.50) | 0.27  (0.24 - 0.29) | 1365.79  (1256.88 - 1569.30) | 6.29  (5.40 - 6.82) |
| n | 81 | 88 | 32 | 29 | 31 |
| cTnT (ng/L) >3 | 5.8  (5.6 - 6.3) | 0.43  (0.39 - 0.50) | 0.28  (0.24 – 0.33) | 1444.10  (1230.35 - 1657.85) | 6.87  (6.27 - 7.46) |
| n | 8 | 6 | 2 | 2 | 2 |
| p-value | 0.85 | 0.43 | 0.83 | 0.94 | 0.37 |

CIMT: carotid intima-media thickness; cTnT: cardiac troponin T; IQR: interquartile range; PWV: pulse wave velocity; RWT: relative wall thickness; TVR: total vascular resistance.

**Supplementary Table S11.** Cardiovascular risk factors, biomarkers and assessment levels in women with previous HDP (n=176), stratified by blood pressure categories (according to ACC/AHA classification) [11] at 1-3 years follow-up. Values are presented as median (min-max). A p-value <0.05 is marked with asterix (*).

| CARDIOVASCULAR BIOMARKERS  1-3 YEARS POSTPARTUM | Normotension (n=118)  SBP <120 and DBP <80 | n | Elevated BP/Hypertension (n=58)  SBP ≥120 and DBP ≥80 mmHg | n | p |
| --- | --- | --- | --- | --- | --- |
| CLINICAL BIOMARKERS |  |  |  |  |  |
| BMI (kg/m^2^) | 23.3 (18.9 - 33.7) | 118 | 26.2 (18.1 - 39.5) | 57 | <0.01* |
| Overweight (BMI ≥ 25 kg/m² and BMI <30 kg/m²) | 28% (33) | 118 | 47% (27) | 57 | 0.01* |
| Obesity (BMI ≥ 30 kg/m²) | 9% (10) | 118 | 17% (10) | 57 | 0.08 |
| Premature CVD in 1st degree relative | 28% (33) | 116 | 38% (22) | 57 | 0.18 |
| Current smoking (yes) | 8% (9) | 117 | 5% (3) | 58 | 0.54 |
| CIRCULATING LIPIDS |  |  |  |  |  |
| LDL cholesterol (mmol/L) | 2.69 (1.10 - 4.78) | 118 | 2.93 (1.64 - 4.86) | 58 | 0.04* |
| HDL cholesterol (mmol/L) | 1.52 (0.71 - 3.03) | 118 | 1.48 (0.80 - 2.44) | 58 | 0.26 |
| Total cholesterol (mmol/L) | 4.45 (2.80 - 6.80) | 118 | 4.65 (3.10 - 7.20) | 58 | 0.09 |
| Non-HDL cholesterol (mmol/L) | 2.81 (1.33 - 5.10) | 118 | 3.08 (1.92 - 5.26) | 58 | 0.03* |
| Triglycerides (mmol/L) | 0.66 (0.21 - 2.55) | 118 | 0.78 (0.32 - 2.80) | 58 | <0.01* |
| KIDNEY FUNCTION |  |  |  |  |  |
| Creatinine (µmol/L) | 62 (25 - 95) | 118 | 64 (47 - 92) | 57 | 0.19 |
| eGFR (ml/min/1.73 m²) | 115.12 (69.11 - 142.92) | 118 | 111.71 (71.38 - 129.29) | 57 | <0.01* |
| eGFR (60-89 ml/min/1.73 m²) | 5% (6) | 118 | 12% (7) | 57 | 0.09 |
| Albumin/creatinine ratio (urine; mg/mmol) | 0.5 (0.0 - 12.2) | 114 | 0.4 (0.1 - 21.2) | 55 | 0.75 |
| Elevated albumin/creatinine ratio (≥3 mg/mmol) | 9% (10) | 114 | 12% (7) | 55 | 0.42 |
| METABOLIC CONTROL |  |  |  |  |  |
| Glucose (mmol/L) | 4.6 (3.3 - 5.5) | 116 | 4.7 (4.1 - 6.3) | 55 | 0.04* |
| Glucose 2 hours after OGTT (mmol/L) | 5.3 (3.1 - 9.9) | 52 | 5.4 (3.2 - 8.0) | 15 | 0.16 |
| HbA1c (%) | 5.0 (4.3 - 5.6) | 53 | 4.9 (4.5 - 5.8) | 22 | 0.35 |
| INFLAMMATORY AND CARDIOVASCULAR BIOMARKERS | | |  |  |  |
| hs-CRP (mg/L) | 0.6 (0.0 - 25.3) | 118 | 1.1 (0.0 - 13.1) | 57 | <0.01* |
| sHLA-G (µmol/L) | 457.96 (30.51 - 1520.54) | 113 | 505.55 (86.99 - 2191.51) | 53 | 0.31 |
| SAA1 (ng/ml) | 2210.51 (200.00 - 85927.65) | 118 | 2128.88 (742.03 - 52011.10) | 57 | 0.33 |
| GDF-15 (ng/L) | 432 (400 - 979) | 113 | 440 (400 - 837) | 55 | 0.69 |
| Cardiac troponin T (ng/L) | 3 (3 - 9) | 114 | 3 (3 - 6) | 55 | 0.17 |
| NT-proBNP (ng/L) | 45 (6 - 235) | 114 | 55 (14 - 177) | 55 | 0.09 |
| CARDIOVASCULAR STRUCTURE AND FUNCTION | |  |  |  |  |
| PWV (m/s) | 6.0 (4.5 - 8.8) | 108 | 6.8 (4.7 - 9.4) | 46 | <0.01* |
| CIMT (mm) | 0.46 (0.35 - 0.68) | 109 | 0.50 (0.37 - 0.89) | 53 | <0.01* |
| RWT | 0.28 (0.20 - 0.40) | 37 | 0.28 (0.21 - 0.38) | 23 | 0.63 |
| TVR (dyne/s/cm^-5^) | 1483.41 (852.88 - 1943.18) | 33 | 1624.74 (1151.52 - 2114.18) | 23 | 0.02* |
| E/e´ ratio | 6.29 (4.21 - 9.07) | 36 | 6.11 (4.22 - 9.38) | 23 | 0.95 |

The Mann–Whitney U test comparing continuous variables and the chi-square test comparing categorical variables between blood pressure levels in the prior HDP group at 1-3 years follow-up. Values for continuous variables are presented as medians (range) and categorical variables are presented as % (n). Normotension (n=142) was defined as SBP<120 mmHg and DBP <80 mmHg (blood pressure categories as defined by The American College of Cardiology/American Heart Association (ACC/AHA) Task Force on Clinical Practice Guidelines [11]). Elevated BP and hypertension (n=58) were defined as SBP ≥120 and DBP ≥80 mmHg [11], as well as women using antihypertensive medication.

BMI: body mass index; BP: blood pressure; CIMT: carotid intima-media thickness; CVD: cardiovascular disease; DBP: diastolic BP; eGFR: estimated glomerular filtration rate; GDF-15: growth differentiation factor 15; HDL: high-density lipoprotein; HDP: hypertensive disorders of pregnancy (including both preeclampsia and gestational hypertension patients); hs-CRP: high sensitivity C-reactive protein; LDL: low-density lipoprotein; NT-proBNP: N-terminal pro-brain natriuretic peptide; OGTT: oral glucose tolerance test; PE: preeclampsia; SAA1: serum amyloid A1; sHLA-G: soluble human leukocyte antigen-G; PWV: pulse wave velocity; RWT: relative wall thickness; SBP: systolic BP; TVR: total vascular resistance.

**References:**

1. Klepp K, Moe K, Angel K, von Lueder TG, Sugulle M, Ziesler C, et al. Cardiovascular phenotype in women 1-3 years after hypertensive pregnancy disorders: Impact of sex-independent and pregnancy-specific risk factors. Pregnancy Hypertens. 2025;43:101404. doi: 10.1016/j.preghy.2025.101404.

2. Naderi N. Practical Cardiology. Second ed. Elsevier; 2022.

3. Devereux RB, Reichek N. Echocardiographic determination of left ventricular mass in man. Anatomic validation of the method. Circulation. 1977;55(4):613-8. doi: 10.1161/01.cir.55.4.613.

4. Reichek N, Devereux RB. Reliable estimation of peak left ventricular systolic pressure by M-mode echographic-determined end-diastolic relative wall thickness: identification of severe valvular aortic stenosis in adult patients. Am Heart J. 1982;103(2):202-3. doi: 10.1016/0002-8703(82)90493-8.

5. Determinants of pulse wave velocity in healthy people and in the presence of cardiovascular risk factors: 'establishing normal and reference values'. Eur Heart J. 2010;31(19):2338-50. doi: 10.1093/eurheartj/ehq165.

6. Mancia G, Fagard R, Narkiewicz K, Redón J, Zanchetti A, Böhm M, et al. 2013 ESH/ESC Guidelines for the management of arterial hypertension: the Task Force for the management of arterial hypertension of the European Society of Hypertension (ESH) and of the European Society of Cardiology (ESC). J Hypertens. 2013;31(7):1281-357. doi: 10.1097/01.hjh.0000431740.32696.cc.

7. Lang RM, Badano LP, Mor-Avi V, Afilalo J, Armstrong A, Ernande L, et al. Recommendations for cardiac chamber quantification by echocardiography in adults: an update from the American Society of Echocardiography and the European Association of Cardiovascular Imaging. J Am Soc Echocardiogr. 2015;28(1):1-39.e14. doi: 10.1016/j.echo.2014.10.003.

8. Patel HN, Miyoshi T, Addetia K, Henry MP, Citro R, Daimon M, et al. Normal Values of Cardiac Output and Stroke Volume According to Measurement Technique, Age, Sex, and Ethnicity: Results of the World Alliance of Societies of Echocardiography Study. J Am Soc Echocardiogr. 2021;34(10):1077-85.e1. doi: 10.1016/j.echo.2021.05.012.

9. Dalen H, Thorstensen A, Vatten LJ, Aase SA, Stoylen A. Reference values and distribution of conventional echocardiographic Doppler measures and longitudinal tissue Doppler velocities in a population free from cardiovascular disease. Circ Cardiovasc Imaging. 2010;3(5):614-22. doi: 10.1161/circimaging.109.926022.

10. Mitter SS, Shah SJ, Thomas JD. A Test in Context: E/A and E/e' to Assess Diastolic Dysfunction and LV Filling Pressure. J Am Coll Cardiol. 2017;69(11):1451-64. doi: 10.1016/j.jacc.2016.12.037.

11. Whelton PK, Carey RM, Aronow WS, Casey DE, Jr., Collins KJ, Dennison Himmelfarb C, et al. 2017 ACC/AHA/AAPA/ABC/ACPM/AGS/APhA/ASH/ASPC/NMA/PCNA Guideline for the Prevention, Detection, Evaluation, and Management of High Blood Pressure in Adults: A Report of the American College of Cardiology/American Heart Association Task Force on Clinical Practice Guidelines. Hypertension. 2018;71(6):e13-e115. doi: 10.1161/hyp.0000000000000065.
